# A Two-Stage Method to Estimate the Contribution of Road Traffic to PM_2.5_ Concentrations in Beijing, China

**DOI:** 10.3390/ijerph13010124

**Published:** 2016-01-13

**Authors:** Xin Fang, Runkui Li, Qun Xu, Matteo Bottai, Fang Fang, Yang Cao

**Affiliations:** 1 Unit of Biostatistics, Institute of Environmental Medicine, Karolinska Institutet, Stockholm 17177, Sweden; xin.fang@ki.se (X.F.); matteo.bottai@ki.se (M.B.); 2 College of Resources and Environment, University of Chinese Academy of Sciences, Beijing 100049, China; 3 State Key Laboratory of Resources and Environmental Information System, Institute of Geographic Sciences and Natural Resources Research, Chinese Academy of Sciences, Beijing 100101, China; 4 Department of Epidemiology and Biostatistics, Institute of Basic Medicine Sciences, Chinese Academy of Medical Sciences & School of Basic Medicine, Peking Union Medical College, Beijing 100005, China; xuqun@ibms.cams.cn; 5 Department of Medical Epidemiology and Biostatistics, Karolinska Institutet, Stockholm 17177, Sweden; fang.fang@ki.se; 6 Clinical Epidemiology and Biostatistics, Faculty of Medicine and Health, Örebro University, Örebro 70281, Sweden

**Keywords:** PM_2.5_ concentration, road traffic contribution, atmospheric dispersion model, generalized additive mixed model

## Abstract

*Background*: Fine particulate matters with aerodynamic diameters smaller than 2.5 micrometers (PM_2.5_) have been a critical environmental problem in China due to the rapid road vehicle growth in recent years. To date, most methods available to estimate traffic contributions to ambient PM_2.5_ concentration are often hampered by the need for collecting data on traffic volume, vehicle type and emission profile. *Objective*: To develop a simplified and indirect method to estimate the contribution of traffic to PM_2.5_ concentration in Beijing, China. *Methods*: Hourly PM_2.5_ concentration data, daily meteorological data and geographic information were collected at 35 air quality monitoring (AQM) stations in Beijing between 2013 and 2014. Based on the PM_2.5_ concentrations of different AQM station types, a two-stage method comprising a dispersion model and generalized additive mixed model (GAMM) was developed to estimate separately the traffic and non-traffic contributions to daily PM_2.5_ concentration. The geographical trend of PM_2.5_ concentrations was investigated using generalized linear mixed model. The temporal trend of PM_2.5_ and non-linear relationship between PM_2.5_ and meteorological conditions were assessed using GAMM. *Results*: The medians of daily PM_2.5_ concentrations during 2013–2014 at 35 AQM stations in Beijing ranged from 40 to 92 μg/m^3^. There was a significant increasing trend of PM_2.5_ concentration from north to south. The contributions of road traffic to daily PM_2.5_ concentrations ranged from 17.2% to 37.3% with an average 30%. The greatest contribution was found at AQM stations near busy roads. On average, the contribution of road traffic at urban stations was 14% higher than that at rural stations. *Conclusions*: Traffic emissions account for a substantial share of daily total PM_2.5_ concentrations in Beijing. Our two-stage method is a useful and convenient tool in ecological and epidemiological studies to estimate the traffic contribution to PM_2.5_ concentrations when there is limited information on vehicle number and types and emission profile.

## 1. Introduction

According to a growing body of epidemiological evidence traffic-related air pollution has been shown to have adverse health impacts. Fine particulate matters with aerodynamic diameters smaller than 2.5 micrometers (PM_2.5_) pose great public health hazards, including higher risks of respiratory diseases, impaired lung function, asthma attacks, cardiovascular diseases, and potentially also premature death [1].

The particulates generated from combustion are more harmful than those generated from other processes, and traffic emissions are estimated to account for up to 50% of combustion-generated particulates in urban areas in developing countries [2]. According to the Ministry of Environmental Protection of China, traffic emissions have become the main source of air pollution in Beijing [3]. Among all air pollutants, PM_2.5_ is of special importance in China due to the rapidly growing number of road vehicles in recent years. By collecting and analyzing aerosol samples of PM_2.5_ and PM_10_ both in summer and winter seasons at different traffic, industrial and residential areas in Beijing, a multisite study found that industrial and motor vehicle emissions, together with coal burning, were the major contributors to the air-borne particulate pollution in Beijing [4].

Although the Beijing Environmental Protection Bureau started monitoring air pollution in 1984, monitoring of PM_2.5_ only started in 2006. Prior to that, PM_2.5_ was mainly used for air pollution research purposes [5]. As a result of increasing demand from the public, since October 2012, Beijing has increased its number of fixed air quality monitoring (AQM) stations from 27 to 35 across the entire municipal area. In addition to carbon dioxide, sulfur dioxide, nitrogen dioxide, ozone and PM_10_, PM_2.5_ has also been included in the air quality evaluations of these AQM stations. A study found that, while burning of coal for power plants is a major source of air pollution across China, vehicle emissions are one of the biggest sources of PM_2.5_ in Beijing, with greater impact than soil dust, fossil fuel combustion, biomass burning and some industrial sources [6]. Although previous studies have clearly shown that the contribution of traffic emissions to total air pollution varies largely with time and space, they were unable to characterize the spatiotemporal features of the traffic-related PM_2.5_ because of limited information on location and time period for air sample collection [5].

Chemical mass balanced receptor models and source-oriented chemical transport models have been used to estimate the contributions of various sources to PM_2.5_, but most of them require the knowledge of the chemical profile of both the emissions of the sources and the air samples of the receptors (*i.e.*, the impacted locations) [7,8]. Although other models such as principal component and factor analyses do not require a priori knowledge of the source profile, application of these models yielded controversial results. For example, the estimated motor-vehicle contribution to PM_2.5_ ranged from 6% in Beijing, China to 53% in Barcelona, Spain [9].

Although traffic emission is the principal source of intra-urban concentration of PM_2.5_, one reason that the direct measurement of motor-vehicle emission may not be feasible in epidemiological studies is that it is usually not possible to track all the vehicles and measure corresponding components of the traffic-pollutant mix in the whole study area [10]. As a result, different surrogates of traffic-related pollution have been used to assess the contribution of road traffic to ambient air pollution. In epidemiological studies, the commonly used surrogate models include geostatistical interpolation [11], land-use regression [12], dispersion [13] and hybrid [14] models. Hybrid models combine personal activity of residents in the study area and exposure data, and incorporate various measurements, therefore better quantify the contribution of traffic on air pollution, against a background concentration of specific regions. However, none of the models has an ideal surrogate to access the emissions from all sources over time and space, posing a significant challenge in disentangling the contribution of road traffic from other sources.

To improve the assessment of traffic-related contributions to PM_2.5_, a promising method is the deployment of a large number of AQM stations in places where concentrations of PM_2.5_ are expected to be highly variable, and with available information on temporal and spatial factors [15]. The intensive air quality data that we collected from 35 AQM stations in Beijing, one of the most populous cities in the world, between 2013 and 2014, provided us a unique opportunity to achieve this purpose. In our paper, we presented a two-stage method using dispersion model and generalized additive mixed model (GAMM) to estimate the contribution of road traffic to PM_2.5_ concentrations in Beijing. We used different types of the AQM stations (described in Material and Methods section) to distinguish the emission sources of PM_2.5_, adjusted for the location of these stations, traffic density and meteorological conditions. In the first stage, a Community Multi-scale Air Quality (CMAQ) based model was built to estimate the contribution of road vehicle emission to PM_2.5_ as a result of dispersion and decay in the areas represented by background stations [16]. In the second stage, a GAMM with meteorological and geographic data was developed to estimate the non-traffic contribution to PM _2.5_ at the rest stations. The traffic contribution to PM_2.5_ was then calculated by subtracting the total PM_2.5_ concentration with non-traffic concentration. The study was approved by the Institutional Review Board of Karolinska Institutet, Sweden.

## 2. Materials and Methods

### 2.1. Data Collection

**Figure 1:**
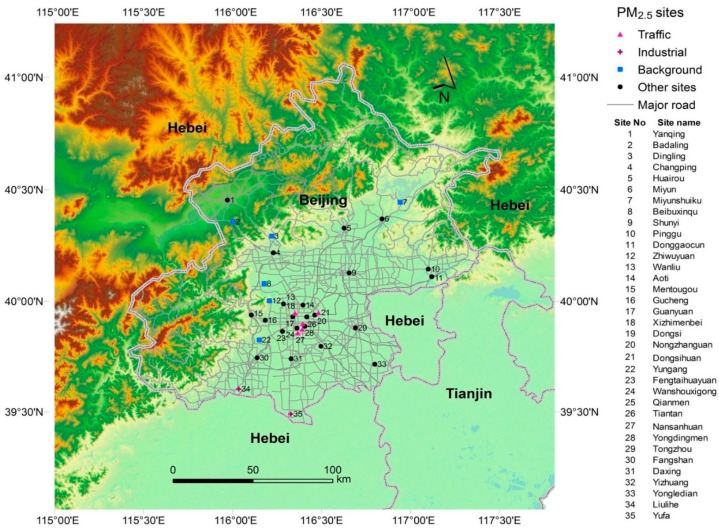
Distribution of 35 Air Quality Mornitoring (AQM) stations in Beijing.

Hourly concentrations of PM_2.5_ were collected from 35 AQM stations in Beijing from 1 January 2013 to 31 December 2014. The AQM stations were installed by the Beijing Municipal Environmental Protection Bureau. The aim of these stations was to assess the air quality under different conditions from the most polluted area with high density of traffic to the least polluted rural areas in Beijing. Thus, air pollution concentrations of these stations vary largely from each other due to the variation of their distances to pollution sources, e.g., traffic emissions and industrial emissions. The distribution of the AQM stations was shown in Figure 1. These stations scattered from the very south to the north of Beijing, from the central urban areas to countryside, covering most of the spatial regions and typical land types. Geographic information of these stations was attained from College of Resources and Environment, University of Chinese Academy of Sciences. According to the Ambient Air Quality Standards and Technical Regulation on Ambient Quality Index of China, 24-hour concentrations of PM_2.5_ and individual air quality index (IAQI) were reported hourly from these stations [17]. The air quality has been classified by Chinese Environmental Protection Agency into six categories, *i.e.*, “Good”, ”Moderate”, ”Unhealthy for Sensitive Groups”, “Unhealthy”, ”Very Unhealthy” and “Hazardous” [17,18]. Duplicated records were first removed from the dataset, and the records with empty or 0 value were treated as missing. The missing rate was 9% and no apparent trend was found for the missing values. In total, 553,877 PM_2.5_ concentration records were collected from the 35 monitoring stations in 730 days in 2013 and 2014. Values greater than 10 times the 75% percentile or smaller than one-tenth of the 25% percentile of all the records were treated as abnormal values and only included in sensitivity analyses.

Daily meteorological data were collected from National Meteorological Information Center of China in the same period, including air temperature, atmospheric pressure, wind speed, wind direction, volume of rainfall and hours of daylight. Five-minute traffic volume and speed data per 30 minutes for four days from eight crossroads in core districts in Beijing were collected by the College of Resources and Environment, University of Chinese Academy of Sciences. The traffic density of the monitoring stations in these districts was characterized by an inverse function of mean road vehicle speed on the main roads [19].

### 2.2. Fitting Spatial Trend of PM_2.5_ Concentration

Historical data and previous findings showed that air pollution was often heavier in the southern part than the northern part of Beijing [20], therefore a three-level generalized linear mixed model (GLMM) was fitted between the geographical Y coordinates (*i.e.*, distance from an AQM station to the southern boundary of Beijing ) of the AQM stations in a rectangular coordinate system and the log transformed PM_2.5_ concentrations (logPM_2.5_). The Y coordinates were used as an independent variable, whereas calendar days and hours of each calendar day were include as random effects in the model.

Because background stations are less but still affected by traffic pollution, and non-traffic portion of PM_2.5_ pollutants is more geographically stable, fitting a regional non-traffic trend in the study area that takes advantage of the background stations is plausible. The final traffic contribution could be calculated by subtracting the non-traffic portion from the total observed concentration. The two-stage method is described in detail below:

#### 2.2.1. Stage 1: Estimating the Traffic Contribution to PM_2.5_ Concentration at Background Stations Using Dispersion Model

Based on the sources of air pollution, we divided the 35 AQM stations into four groups: six background stations, five traffic stations, two industrial stations and 22 other stations. The six background stations are located at Dingling, Yungang, Beibuxinqu, Zhiwuyuan, Miyunshuiku and Badaling, located far away from both urban areas and industrial areas and had few direct traffic and industrial emissions. The air pollution at these stations is mainly from dispersed pollutants, and the PM_2.5_ concentration of these stations can be regarded as the background pollution concentration in each region. The five traffic stations include Dongsihuan, Nansanhuan, Qianmen, Xizhimenbei and Yongdingmen, which are less than 10 meters away from the main roads of Beijing, where the PM_2.5_ concentration mainly derives from traffic emissions. The two industrial stations include Liulihe and Yufa which are located at the southern boundary between Beijing and Heibei Province where the PM_2.5_ concentration is mainly caused by local industrial emission and dispersion. For traffic stations, the PM_2.5_ pollutants were mainly from vehicle emissions. The total PM_2.5_ concentration of the five stations was considered as a surrogate of the PM_2.5_ from traffic emissions. Two industrial stations close to the southern boundary of Beijing are located near to an industrial area of Hebei Province. The PM_2.5_ concentrations of these two stations are therefore treated as a surrogate of the industrial emissions.

Based on the Hybrid Single-Particle Lagrangian Integrated Trajectory (HYSPLIT) model, backward trajectories were used to track the transport corridors that are regarded as a “region of influence” *i.e.*, the five traffic stations and two industrial stations in our study [21]. Both traffic and industrial stations were considered as PM_2.5_ sources of the six background stations because no other major PM_2.5_ contributors were found near the background stations. Because dispersion processes are largely additive, PM_2.5_ pollution at every station is supposed to be consisted of remaining from daily deposition and dispersion from different emission source points, *i.e.*, traffic, factories and other sources (such as household cooking and coal burning) [22,23]. For the background stations, PM_2.5_ contribution other than traffic and industrial dispersed is considered as station-specific background PM_2.5_ concentration.

Since distance also played an important role for pollutant dispersion, the inversed value of distance from source stations (*i.e.*, traffic stations and industrial stations) to the receptor stations (*i.e.*, background station) was put as a weight of dispersion factor.

According to the Community Multiscale Air Quality (CMAQ) model, all emissions are assumed to be instantaneously well-mixed and have their own atmospheric lifetime [24]. Therefore a strong daily dependence is expected on consecutive days. We assume that in the condition of wind, PM_2.5_ can partly linger for at least one day [25]. Analogously, we built a dispersion model as shown in model (1), in which the PM_2.5_ concentration was presented as a summation of traffic dispersion, industrial dispersion and the remaining from daily deposition. Because the pollution carried by wind had a strong positive relationship with the wind flux, a power function was used to fit dispersion effect. The daily deposition of pollution with interaction of wind was fitted by the exponential function:

(1) $${\hat{C}}_{p\left(t\right)}=[k_{1}\;C_{p\left(t-1\right)}+k_{2}\times \frac{1}{\sqrt{D_{ind_{p}}}}\times C_{ind\left(t\right)}\times {\left({\hat{W}}_{ind\left(t\right)}/W_{avg}\right)}^{k_{3}}+k_{4}\times \frac{1}{\sqrt{D_{traffic_{p}}}}\times C_{traffic\left(t\right)}\times \left({\hat{W}}_{traffic\left(t\right)}/W_{avg}{)}^{k_{3}}\right]\times e^{-k_{5}\times W_{\left(t\right)}}$$

In model (1): ${\hat{C}}_{p\left(t\right)}$denotes the expected PM_2.5_ concentration at station p on day *t*. $C_{p\left(t-1\right)}$ denotes the observed PM_2.5_ concentration on day *t*-1; $D_{ind_{p}}$ represents the average distance from station *p* to industrial stations; $C_{ind\left(t\right)}$ denotes the observed PM_2.5_ concentration of industrials stations on day *t*; and $D_{traffic_{p}}$ represents the average distance from station *p* to traffic stations;$\;C_{traffic\left(t\right)}$ denotes the observed PM_2.5_ concentration of traffic stations on day *t*; ${\hat{W}}_{ind\left(t\right)}$ denotes the summation of valid flux of wind from industrial stations and ${\hat{W}}_{traffic\left(t\right)}$ means the summation of valid flux of wind from traffic stations on day *t*; $W_{avg}$ is the average wind speed of the year; $W_{\left(t\right)}$ is the maximum wind speed on day $t;\text{and}\;k_{1},\cdot \cdot \cdot ,k_{5}$ are the parameters to be estimated by Levenberg-Marquardt and global minimum algorithm till their convergence [26].

In model (1), $k_{1}\times \;C_{p\left(t-1\right)}$ describes the residual concentration on last day’s pollution; $k_{2}\;\times \frac{1}{\sqrt{D_{ind_{p}}}}\times C_{ind\left(t\right)}\times {\left({\hat{W}}_{ind\left(t\right)}/W_{avg}\right)}^{k_{3}}$ illustrates PM_2.5_ concentration from industrial stations by dispersion; $k_{4}\times \frac{1}{\sqrt{D_{traffic_{p}}}}\times C_{traffic\left(t\right)}\times {\left({\hat{W}}_{traffic\left(t\right)}/W_{avg}\right)}^{k_{3}}$ illustrates the traffic PM_2.5_ concentration from traffic stations by dispersion. The sum of these three components is allowed to decay with increasing wind by the factor $e^{-k_{5}\times W_{\left(t\right)}}$.

According to model (1), if $W_{\left(t\right)}=0$, which means there were no wind/dispersion at all, the model (1) reduces to its simplest format:

(2) $${\hat{C}}_{p\left(t\right)}=k_{1}\times \;C_{p\left(t-1\right)}$$

To estimate the parameters in model (1), we obtained following data: daily PM_2.5_ concentration of the six background stations$\;C_{p\left(t\right)}$, (*p* = 1, …, 6); daily PM_2.5_ concentration of the two industrial stations $C_{j\left(t\right)}$.(*j* = 1, 2); daily PM_2.5_ concentration of the five traffic stations $C_{k\left(t\right)}$.(*k* = 1, …, 5); distance from each background station to each industrial station $\;D_{ind_{jp}}$; distance from each background station to each traffic station $\;D_{traffic_{kp}}$; daily maximum wind speed $W_{\left(t\right)}$; direction of daily maximum wind ${\vec{m}}_{w}$, which is given in 16 compass points clockwise from the *Y* coordinate.

Let ${\vec{m}}_{w}$ denote the unit vector of wind direction, and *θ* be the degree of direction from source station to receptor station *p* deviating from the *Y* coordinate, then:

(3) $${\vec{m}}_{w}=-\left(\cos\theta ,\sin\theta \right)$$

Let ${\vec{R}}_{p}$ denote the direction vector from the centroid of source stations to the receptor station *p*, then the summation of valid wind flux ${\hat{W}}_{\left(t\right)}$ to station *p* is given as:

(4) $${\hat{W}}_{\left(t\right)}={\vec{W}}_{\left(t\right)}\times \frac{\left({\vec{R}}_{p\left(t\right)}\times {\vec{m}}_{w}\right)}{\left|\left({\vec{R}}_{p\left(t\right)}\times {\vec{m}}_{w}\right)\right|}$$

where ${\vec{W}}_{\left(t\right)}$ is the vector value of $W_{\left(t\right)}$.

In our projection, we limited the minimum pollution brought by wind to nonnegative value, thus:

(5) $${\hat{W}}_{\left(t\right)}=\max\left({\hat{W}}_{\left(t\right)},0\right)$$

Based on model (1), the daily traffic contribution to PM_2.5_ at background stations can be calculated as:

(6) $$T_{p\left(t\right)}\%=\frac{k_{4}\times \frac{1}{\sqrt{D_{traffic_{p}}}}\times C_{traffic\left(t\right)}\times {\left(\frac{{\hat{W}}_{traffic\left(t\right)}}{W_{avg}}\right)}^{k_{3}}\times e^{-k_{5}\times W_{\left(t\right)}}}{C_{p\left(t\right)}}\times 100\%$$

where $T_{p\left(t\right)}\%$ is estimated percentage of daily traffic contribution to total PM_2.5_ concentration at background stations. Meanwhile, the expected daily non-traffic contribution ${{NT}_{p\left(t\right)}}^{*}$ can be calculated as:

(7) $${{NT}_{p\left(t\right)}}^{*}=C_{p\left(t\right)}\times \left(1-T_{p\left(t\right)}\%\right)$$

#### 2.2.2. Stage 2: Estimating Non-Traffic Contribution to PM_2.5_ Concentrations at Non-Background Stations Using GAMM

**Figure 2:**
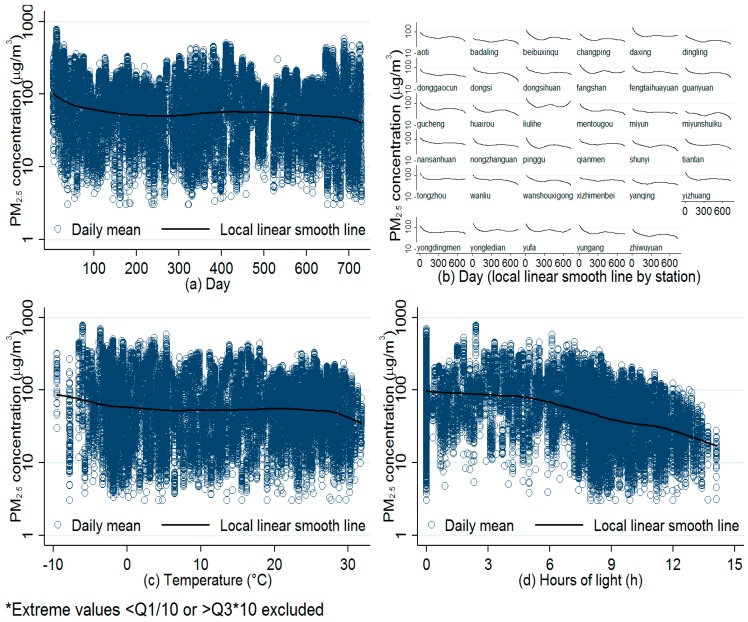
Relationship between daily mean PM_2.5_ concentrations and day (**a**) at all stations and (**b**) by stations; relationship between daily mean PM_2.5_ concentrations and (**c**) daily mean temperature and (**d**) daily hours of light.

A GAMM was fitted between log transformed daily non-traffic PM_2.5_ concentration $\text{log}NT_{p\left(t\right)}$ and Y coordinates (*Y_p_*) for the background stations. Because there were apparent nonlinear relationship between daily PM_2.5_ concentration and day (*t*) (Figure 2a), humidity, temperature and atmospheric pressure (atmos) (Figure 2c), we used B-spline penalized by discrete penalties as additive smoothing function in the GAMM. Ten knots per year were set for day, five for humid, five for temperature and four for atmos, respectively. Numbers of knots were determined by minimizing Akaike Information Criterion (AIC) [27]. Besides the *Y_p_*, our preliminary analyses suggested linear associations between daily PM_2.5_ concentration and wind speed (*Wind*_(*t*)_), rain volume (*Rain*_(*t*)_) and hours of daylight (*Light*_(*t*)_) (Figure 2d), they were included as covariates in the GAMM in addition to *Y_p_*. Day of week (*DOW*_(*t*)_) and direction of daily maximum wind speed (*Max_wind_dir*_(*t*)_) were included as factor variables in the model. In addition, considering the intra-cluster correlation of PM_2.5_ concentration within stations, we included a random effect for stations in the model (Figure 2b). The selection of explanatory variables was also decided by a top-down rule [28]. The model was run by stepwise approach and generalized cross-validation (GCV) criterion [29]. The final GAMM is:

(8) $$\begin{array}{cc} log{\left(NT_{p\left(t\right)}\right)}^{\;*} & =\beta_{0}+\beta_{1}\times Y_{p}+\beta_{2}\times Wind_{\left(t\right)}+\beta_{3}\times Light_{\left(t\right)}+\beta_{4}\\ & \times Rain_{\left(t\right)}+\beta_{5}\times Max_{\_}{wind}_{\_}{dir}_{\left(t\right)}+\beta_{6}\times DOW_{t}\\ & +s\left(t,\;k=10\;per\;year\right)+s\left(temperature_{\left(t\right)},\;k=5\right)\\ & +s\left(humid_{\left(t\right)},\;k=5\right)+s\left(atmos_{\left(t\right)},\;k=4\right)+\mu \times Z_{p} \end{array}$$

where $log{\left(NT_{p\left(t\right)}\right)}^{\;*}$ is expected log transformed non-traffic PM_2.5_ concentration; βs are parameters to be estimated; s(.)s are additive smoothing functions which illustrate the effects of day, temperature and humidity on non-traffic concentrations; *Z_p_* is a random intercept for station *p*.

Log transformed non-traffic PM_2.5_ concentrations at non-background station *q*, $log{\left(NT_{q\left(t\right)}\right)}^{\;*}$, were then predicted using the GAMM fitted in model (8). The estimated contribution of road traffic to PM_2.5_ contribution at non-background station *q*, $T_{q\left(t\right)}\%$, was calculated as observed PM_2.5_ concentration deducted by estimated non-traffic PM_2.5_ concentration:

(9) $$T_{q\left(t\right)}\%=\frac{C_{q\left(t\right)}-e^{\text{log}{\left(NT_{q\left(t\right)}\right)}^{\;*}}}{C_{q\left(t\right)}}\times 100$$

**Figure 3:**
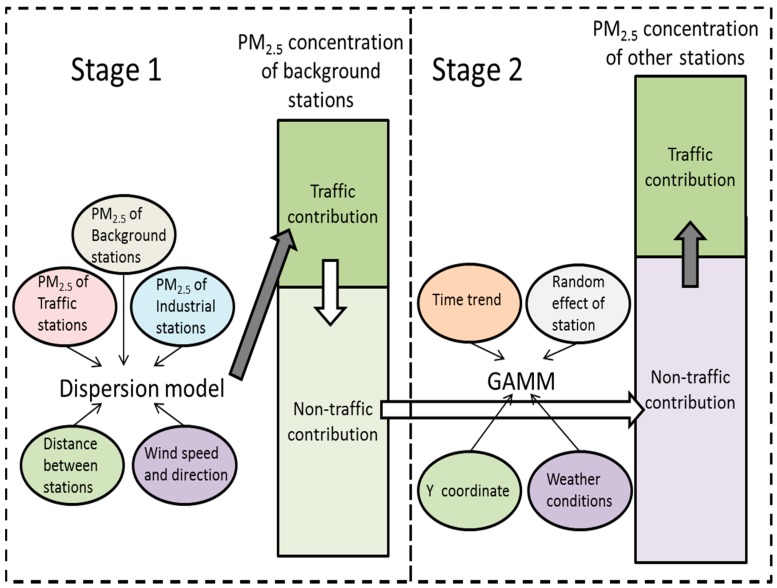
Process of estimating traffic contribution to PM_2.5_ concentration at background AQM stations and other stations.

The whole process of the method is shown in Figure 3. The parameters for dispersion model were estimated in software 1stOpt [26]. GLMM was fitted in Stata 13.1 and GAMM was fitted in R 3.2.2 using mgcv package.

## 3. Results

PM_2.5_ concentrations from the 35 AQM stations and meteorological conditions during 2013–2014 in Beijing are shown in Table 1 and Table 2. The medians of daily PM_2.5_ concentration of the 35 stations ranged from 40 to 92 μg/m^3^. The means of daily PM_2.5_ concentration ranged from 63 to 112 μg/m^3^, higher than 55.4 μg/m^3^ as reported by Yu *et al.* in 2013 [30]. The average PM_2.5_ concentration was almost four times the U.S. Environmental Protection Agency standard (15 μg/m^3^) [31]. In general, background stations had lower whereas traffic stations and industrial stations had higher PM_2.5_ concentrations than the other stations located in the same districts.

**Figure 4:**
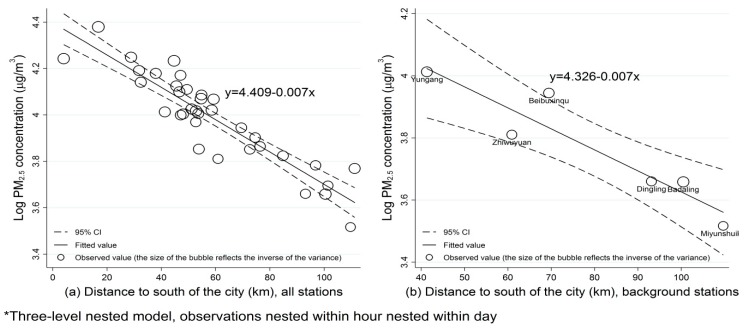
Relationship between Y coordinate (distance to the south of the city) and log transformed PM_2.5_ concentrations at (**a**) all stations and (**b**) background stations.

There was a significant linear relationship between Y coordinates and log transformed PM_2.5_ concentrations both in all stations and in background stations (Figure 4), supporting our assumption that PM_2.5_ concentration followed an exponential decline function on distance. The Y coordinates could explain more than 80% variation of log transformed annual average PM_2.5_ concentrations in all stations. The closer a station was to the south border of the southern industrial area, the heavier the pollution level it had.

The optimal estimation of the parameters and fitness of the model was shown in Table 3. The dispersion model can explain more than 60% variation of the daily PM_2.5_ concentration of the background stations. The unexplained variation might on the other hand be due to temporal trend and meteorological conditions and was modeled in the GAMM later.

**Table 1:** PM_2.5_ concentrations and Y coordinates of 35 AQM stations.

Stations	PM_2.5_ (μg/m^3^)				Y Coordinate (km)
	Mean	P25	Median	P75	
**Background stations**					
Badaling	64.8	17.0	40.0	91.0	100.47
Beibuxinqu	86.5	24.2	62.0	122.7	69.47
Dingling	71.2	15.0	45.0	101.0	93.12
Miyunshuiku	63.4	13.0	40.3	91.0	109.68
Yungang	90.0	28.0	65.0	125.0	41.32
Zhiwuyuan	79.7	19.0	56.0	112.7	60.91
**Traffic stations**					
Dongsihuan	97.5	29.0	71.0	135.0	54.82
Nansanhuan	106.6	36.2	81.0	147.0	44.70
Qianmen	100.0	31.0	76.6	138.8	49.45
Xizhimenbei	92.8	29.0	68.3	127.2	54.66
Yongdingmen	98.0	31.0	73.0	135.1	46.62
**Industrial stations**					
Liulihe	122.2	44.0	92.0	169.0	16.81
Yufa	109.6	38.0	79.8	148.0	4.06
**Other stations**					
Aoti	89.8	27.0	67.0	125.0	58.61
Changping	78.0	19.0	53.0	111.0	84.81
Daxing	106.9	35.0	79.0	147.0	31.81
Donggaocun	79.3	22.0	58.0	113.0	72.61
Dongsi	90.4	25.2	66.5	128.0	52.71
Fangshan	101.2	33.0	75.8	140.8	32.43
Fengtaihuayuan	99.7	31.0	74.1	139.0	45.53
Guanyuan	88.4	27.0	65.5	123.4	52.82
Gucheng	90.0	28.0	67.5	125.0	51.16
Huairou	76.1	19.0	52.9	108.0	96.85
Mentougou	79.2	22.0	55.4	111.0	53.85
Miyun	71.9	17.5	49.0	100.0	101.39
Nongzhanguan	91.3	26.4	66.0	126.0	53.63
Pinggu	80.8	23.0	57.0	111.0	76.40
Shunyi	84.8	22.0	61.0	121.0	74.58
Tiantan	89.0	27.0	66.4	125.2	48.00
Tongzhou	105.7	33.2	79.3	144.0	47.08
Wanliu	93.6	29.8	69.5	130.1	59.28
Wanshouxigong	91.2	26.0	68.0	128.0	47.13
Yanqing	72.0	20.0	49.5	102.0	111.24
Yizhuang	105.3	34.2	78.9	144.0	37.93
Yongledian	111.8	38.7	81.7	149.8	28.87
**Total**	90.0	25.2	65.0	125.5	59.13

P25: the 25th percentile; P75: the 75th percentile.

**Table 2:** Meteorological conditions in Beijing.

Meteorological Conditions	Mean	P25	Median	P75
Temperature (°C)	13.4	3.2	14.3	23.7
Humid (%)	53	38	53	68
Atmospheric pressure (hPa)	1012.5	1004.2	1012.7	1021.1
Wind speed (m/s)	2.1	1.5	1.9	2.5
Hours of light (h)	6.5	2.4	7.8	9.6
Rain volume (mm) *	15.6	-	-	-

* Because 81% of days had no rain, P25, median and P75 are 0.

**Table 3:** Parameters of dispersion model for PM_2.5_ concentrations.

Parameter	Value
*k*_1_	0.7553
*k*_2_	31.6683
*k*_3_	0.2079
*k*_4_	14.8340
*k*_5_	0.1591
Root-mean-square error	43.4203
R	0.7981
R-square	0.6370
Coefficient of determination (adjusted)	0.6171

Based on Equation (6), the road traffic contribution to PM_2.5_ concentration of the background stations is shown in Table 4. The contributions ranged from 17.2% in Yungang to 25.3% in Zhiwuyuan.

**Table 4:** Contribution (%) of road traffic to PM_2.5_ concentrations of background stations.

Station	Mean (%)	95% Confidence Interval (%)
Badaling	20.5	(18.7, 22.2)
Beibuxinqu	19.6	(18.1, 21.1)
Dingling	20.9	(19.2, 22.6)
Miyunshuiku	21.8	(19.5, 24.1)
Yungang	17.2	(15.5, 18.8)
Zhiwuyuan	25.3	(23.3, 27.3)

**Figure 5:**
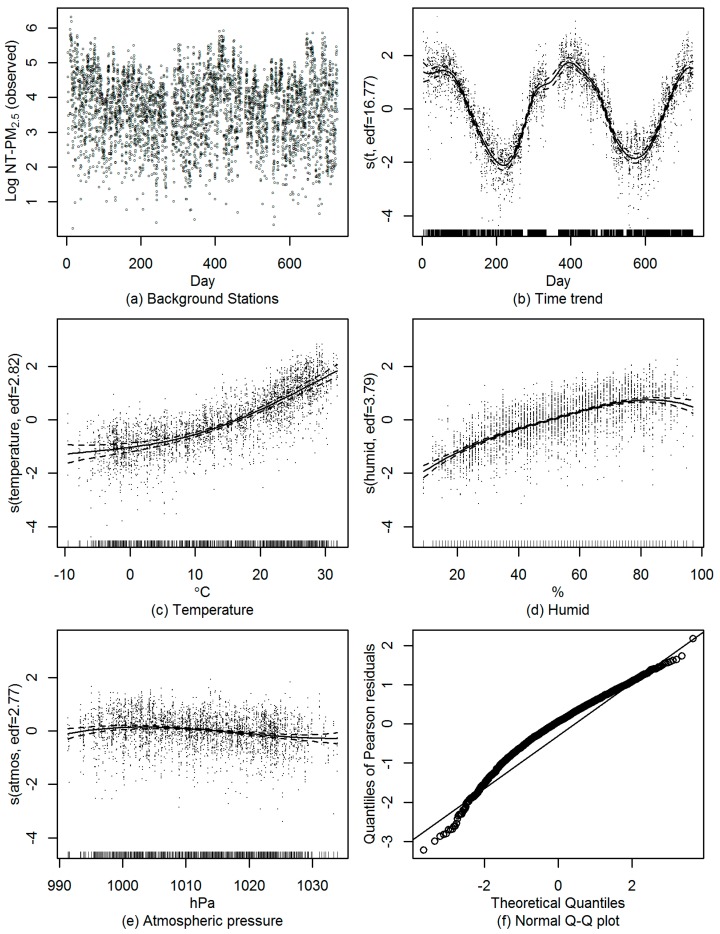
Diagnostic plots of GAMM on non-traffic PM_2.5_ concentrations at background stations: (**a**) time trend of log transformed non-traffic PM_2.5_ concentrations; (**b**) partial regression smooth curve of day with residuals; (**c**) partial regression smooth curve of temperature with residuals; (**d**) partial regression smooth curve of humid with residuals; (**e**) partial regression smooth curve of atmospheric pressure with residuals; (**f**) Q-Q plot of Pearson residuals.

**Figure 6:**
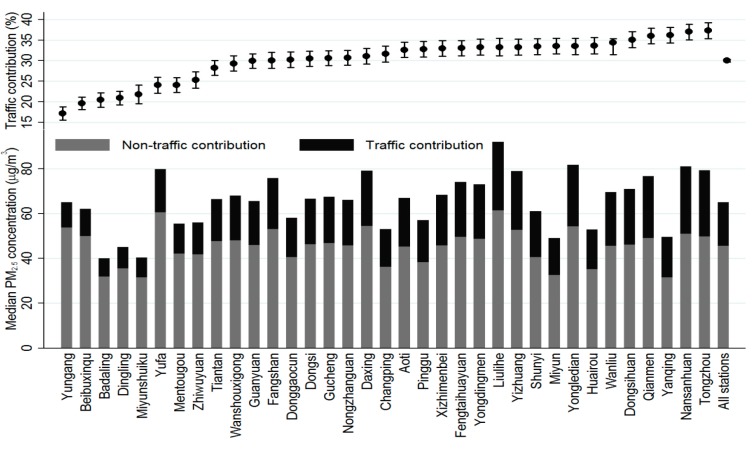
Contribution (%) of road traffic to median PM_2.5_ concentrations by stations in Beijing, 2013–2014.

The estimations of parameters and the approximate test of smoothing of GAMM are shown in Table 5 and Table 6. All coefficients of the linear components and the smooth terms are significant at α = 0.05 level. The result is also in line with the fact that increasing pollution dilution was expected to be associated with greater wind speed and rain volume. According to Yu *et al.* [30], average PM_2.5_ concentration during the days with wind speed higher than 2 m/s was 13% lower than those during the days with weaker wind. Average PM_2.5_ concentration during the rainy days was 21% lower than those during the days without rain. But it is interesting that hours of daylight were negatively associated with the PM_2.5_ concentration. This may be partly due to low dispersion rate during days with fewer daylight hours (usually in hazy and cloudy days) and accelerated accumulation of pollutants. The partial regression smooth plots (Figure 5b–e) and normal Q-Q plot of Pearson residual (Figure 5f) showed a good fit of GAMM. Based on Equation (9), the traffic contribution to PM_2.5_ concentration of other stations is shown in Table 7. The absolute and relative contributions of road traffic to PM_2.5_ concentrations of all stations were summarized in Figure 6. The average annual contribution of road traffic to PM_2.5_ concentration ranged from 17.2% to 37.3% with a mean contribution 30%. The highest contribution was found in busy road areas, and the contribution in traffic-related stations is about 14% higher than those in rural areas.

Because there were no PM_2.5_ values lower than one-tenth of the 25% percentile and only 5% values were higher than 10 times the 75% percentile, the estimated contributions changed little when including these abnormal values in sensitivity analysis (results not shown).

**Table 5:** Parametric coefficients of GAMM (*n* = 3593).

Independent Variable	Estimate	Std. Error	*t* Value	95% Confidence Interval
(Intercept) ***	4.5353	0.1544	29.374	(4.2327, 4.8380)
Y coordinate ***	−0.0063	0.0017	−3.817	(−0.0096, −0.0031)
Wind direction(2) *	0.1358	0.0646	2.103	(0.0092, 0.2624)
Wind direction(3)	0.0246	0.0534	0.461	(−0.0801, 0.1294)
Wind direction(4)	−0.0537	0.0617	−0.871	(−0.1746, 0.0672)
Wind direction(5)	0.0795	0.0719	1.106	(−0.0614. 0.2203)
Wind direction(6)	−0.0738	0.0697	−1.059	(−0.2103, 0.0627)
Wind direction(7) *	−0.2143	0.0905	−2.369	(−0.3917, −0.0370)
Wind direction(8)	0.1302	0.1006	1.294	(−0.0669, 0.3272)
Wind direction(9)	0.0547	0.0611	0.895	(−0.0651, 0.1745)
Wind direction(10) **	0.1480	0.0520	2.845	(0.0460, 0.2499)
Wind direction(11) ***	0.2080	0.0507	4.103	(0.1086, 0.3073)
Wind direction(12) **	0.2481	0.0805	3.084	(0.0904, 0.4059)
Wind direction(13)	0.0634	0.0928	0.684	(−0.1184, 0.2453)
Wind direction(14) *	0.1632	0.0678	2.408	(0.0304, 0.2960)
Wind direction(15)	0.1002	0.0688	1.456	(−0.0347, 0.2351)
Wind direction(16) **	0.1788	0.0601	2.976	(0.0611, 0.2965)
Day of week (2)	−0.0007	0.0405	−0.017	(−0.0800, 0.0786)
Day of week (3)	0.0186	0.0395	0.472	(−0.0587, 0.0960)
Day of week (4)	−0.0009	0.0410	−0.023	(−0.0813, 0.0794)
Day of week (5)	0.0445	0.0408	1.091	(−0.0354, 0.1244)
Day of week (6)	0.0558	0.0400	1.396	(−0.0226, 0.1342)
Day of week (7)	−0.0366	0.0409	−0.894	(−0.1168, 0.0437)
Wind speed *	−0.0402	0.0175	−2.290	(−0.0746, −0.0058)
Hour of light ***	−0.0558	0.0039	−14.404	(−0.0633, −0.0482)
Rain volume ***	−0.0012	0.0002	−6.406	(−0.0015, −0.0008)

*** *p* < 0.001; ** *p* < 0.01; * *p* < 0.05.

**Table 6:** Approximate significance of smooth terms.

	Effective Degree of Freedom (EDF)	F
*s*(t) ***	16.771	64.34
*s*(temperature) ***	2.816	99.28
*s*(humid) ***	3.787	263.91
*s*(atmos) ***	2.767	13.77

*** *p* < 0.001.

**Table 7:** Contribution (%) of road traffic to PM_2.5_ concentrations of other stations.

Station	Mean (%)	95% Confidence Interval (%)
Aoti	32.6	(30.8, 34.5)
Changping	31.6	(29.7, 33.5)
Daxing	31.1	(29.2, 33.0)
Donggaocun	30.2	(28.3, 32.1)
Dongsi	30.5	(28.6, 32.3)
Dongsihuan	35.1	(33.2, 37.0)
Fangshan	30.0	(28.1, 32.0)
Fengtaihuayuan	33.1	(31.2, 34.9)
Guanyuan	29.9	(28.1, 31.6)
Gucheng	30.6	(28.8, 32.4)
Huairou	33.6	(31.6, 35.6)
Liulihe	33.3	(31.2, 35.4)
Mentougou	24.1	(22.3, 25.9)
Miyun	33.5	(31.6, 35.4)
Nansanhuan	37.0	(35.1, 38.8)
Nongzhanguan	30.7	(28.9, 32.5)
Pinggu	32.8	(30.9, 34.7)
Qianmen	36.0	(34.1, 37.9)
Shunyi	33.4	(31.5, 35.3)
Tiantan	28.2	(26.4, 30.0)
Tongzhou	37.3	(35.3, 39.2)
Wanliu	34.4	(32.6, 36.2)
Wanshouxigong	29.3	(27.5, 31.2)
Xizhimenbei	33.0	(31.1, 34.9)
Yanqing	36.2	(34.3, 38.1)
Yizhuang	33.3	(31.4, 35.2)
Yongdingmen	33.3	(31.5, 35.2)
Yongledian	33.5	(31.5. 35.4)
Yufa	24.1	(22.1, 26.0)
All stations *	30.0	(29.7, 30.3)

* Including 6 background stations.

## 4. Discussion

Exhaust emissions due to road traffic are known to make a large contribution to total PM_2.5_ concentrations in urban areas [32,33,34,35] and exposure to PM_2.5_ from vehicular emissions has been demonstrated to have a negative impact on human health [36,37,38,39,40]. An improved understanding of the traffic-related contribution to PM_2.5_ is therefore vital for conducting source apportionment and health effect studies. Due to rapid economic and industrial development and urbanization in the past few decades, energy consumption and the number of motor vehicles are rapidly escalating in China [41]. As the capital of China, Beijing has witnessed a devastating increase in air pollution in the past decades. To develop effective PM_2.5_ reduction strategies, major sources of PM_2.5_ and contributions from each source need to be understood thoroughly. A recent study claimed that vehicles had limited contribution to atmospheric particulate pollution in Beijing [42], and had since caused the public to question the governmental policy in limiting car use. The study presented PM_2.5_ concentrations in all seasons in Beijing and concluded that vehicle emissions accounted for less than 4% of the total PM_2.5_ [42], much smaller than the previous estimates of the Chinese Environmental Protection Agency or as reported by other studies [43,44,45,46]. Other studies using the same data sources suggested however that vehicle contribution to PM_2.5_ in Beijing could vary between 10% and 50% [47,48].

Quantifying traffic-related contribution to PM_2.5_ requires the compilation of detailed traffic data according to time and space, including, for example, traffic counts, vehicle types, travel speeds, fuel types, and emission controls [9]. Receptor models and air-quality dispersion models have been used to assess the contribution of different types of sources, including motor vehicles, to ambient pollution in urban and rural areas [49]. Traditionally, source apportionment estimation methods [50] such as chemical mass balance (CMB) [51] or positive matrix factorization (PMF) have been applied to analyze the contribution of pollutant source. Air mass trajectory analysis is also a useful tool for detecting the direction and location of sources for various air pollutants as a PM_2.5_ forecast model [52]. However, these models heavily rely on the accuracy of source profile information. Some other models were also commonly used, mainly including source apportionment model [53], land use regression model and Gaussian dispersion model [54,55,56]. However, the limited numbers of roadside monitors have made it difficult to catch the geographical variation in motor-vehicle emissions. Resource requirements for collecting these data can be prohibitive and have led to the use of source-oriented dispersion based models [57], meteorological-chemical transport based models [58] and observation-based statistical models [59].

In our study, we developed a two-stage method to estimate the traffic-related contribution to PM_2.5_ concentration that utilized the air-quality data from different types of AQM stations. This method combined atmospheric chemistry dispersion model and statistical GAMM model, and simplified the mathematical algorithm by omitting the detailed traffic-related information, e.g., types, number and density of vehicles, and incorporated the temporal trend of PM_2.5_ concentration in a more precise way. We collected hourly PM_2.5_ data at 35 monitoring stations to estimate the road traffic contributions to PM_2.5_ concentrations. The results revealed that 17.2%–37.3% of PM_2.5_ might be attributable to traffic emissions. Compared to the results released by Beijing Municipal Environmental Protection Bureau (22%–30%) [60], our reported contribution is higher and may partly be due to the rapid increase of traffic volume and decrease of industrial and coal burning emissions in recent years in Beijing [61].

Usually, the estimation of traffic-related emission relies on the analysis of road side measurements correcting for background concentrations [62]. In our study, we carefully defined the components of PM_2.5_ concentration of background stations from two major sources, *i.e.*, traffic emission and industrial sources. Considering the complex components of the traffic related PM_2.5_ source at the traffic stations and industrial stations, relative to the background stations, we modeled the non-traffic PM_2.5_ concentration for all stations using GAMM. The results from previous studies using particulate matter source apportioning and Comprehensive Air Quality Model with Extensions (CAMx) revealed that the maximum level of uncertainty for secondary production was low (6%), hence the application of an additive linear relationship was considered reliable [63,64].

In our dispersion model, the coefficients $k_{3},k_{4},k_{5}$ determine the precision of the estimated traffic contribution to PM_2.5_. We made simulation using different $k_{3},k_{4},k_{5}$ settings for the purpose of sensitivity analysis. The results showed that a 20% deviation in $k_{3},k_{4},\text{or}\;k_{5}$ would result in <7% change in the estimated traffic contribution. It indicated that our dispersion model was robust regarding the variation of the estimates of different parameters.

In order to avoid over-fitting or under-fitting, frequent in GAMM, we used penalized B-splines (P-splines). The P-spline approach controls the coefficients of the smooth function for which a certain penalty term is specified. In this approach, the crucial point is the selection of smoothing parameter. We tested the residual of the model and the scatter plots showed a clear homogeneity around smoothing curves with no specific trend (Figure 5b–e). In our model, the geographical variations were efficiently explained by Y coordinators. A few meteorological variables were selected in the models as previously suggested [65,66,67].

Our study has several strengths. First, most of the previous researches were performed in the United States or Europe, while reliable information from Africa, Asia and South America is lacking. Our study provides important evidence to fill in this information gap and offers an opportunity to develop enhanced methods for quantification of the contribution of traffic emission to air pollution. Second, the two-stage method predicted the background pollution instead of traffic emissions directly. In this case, the residual of the first dispersion model could be further decomposed in the GAMM and the unknown non-linear relationships and temporal autocorrelation were modeled using smoothing functions. Third, although existing dispersion models can give an approximate estimation of traffic emissions based on a big database, they need rich information in terms of vehicle types and fuels, traffic stop-and-go-driving situations, average speed and traffic density, *etc.* [68]. Moreover, the advanced Gaussian dispersion model also requires more complicated 3-dimensional meteorological and location information, making it unfeasible to adapt in less developed countries and regions. Our simplified dispersion model, on the other hand, needs less traffic and geographic data and applies simpler estimation algorithm, and therefore increases flexibility and feasibility of usage. In such context, it is a convenient tool on operational basis for estimating traffic contribution to PM_2.5_ over a region with moderate number of AQM stations. Lastly, because of the limited number of AQM stations available, previous estimates of traffic contribution to PM_2.5_ were mainly based on GAM that might not precisely reflect the variation between stations and correlation within stations in areas with various land use types [69]. The results of such studies were consequently very sensitive to the location of monitoring stations. However, the use of widespread AQM stations and intensive air quality data collected in our study made it possible to involve the different type of stations as a random factor in the mixed effect model that may sufficiently reflect the variation of contribution over a wide region.

Our study also had some limitations. Given the complexity of pollution sources and dynamic dispersion mechanisms, our simplified dispersion model only took into account industrial and traffic emissions, whereas it combined all other pollution sources as a whole. As a result, our method might have led to an overestimation of the traffic contribution. Although we examined the influence of daily average vehicle speed on PM_2.5_ concentrations at five traffic stations and found no statistically significant association, this variable was not included in the GAMM since such information was not available for other stations. Finally, we did not consider some indirect sources from vehicles, such as tire type and asphalt roads that may also increase PM_2.5_ concentration [70]. Future efforts are needed to compare methods using direct traffic emission measurements with our simplified indirect method. We also admit that the predictability of our models is not high and the accuracy of the estimated contributions needs to be assessed by further studies.

## 5. Conclusions

We developed a two-stage method to estimate the traffic contribution to daily PM_2.5_ concentrations in Beijing using hourly PM_2.5_ concentration data, daily meteorological data and geographic information collected at 35 AQM stations in Beijing between 2013 and 2014. Our results showed that traffic emissions accounted for a substantial share of total PM_2.5_ concentrations, ranging from 17% at rural stations to 37% at stations close to busy roads. Our estimates were not only comparable to reports from the Beijing Municipal Environmental Protection Bureau but also reflected the spatial and temporal trends of traffic contribution in a large area. Lacking complete direct measurements of traffic emissions throughout the study area, this method fully utilized the characteristics of different station types. Our method is a useful and feasible tool in ecological and epidemiological studies to estimate the burden of PM_2.5_ derived from road traffic when there is no sufficient traffic-related information.
